# Classification Algorithms for Brain Magnetic Resonance Imaging Images of Patients with End-Stage Renal Disease and Depression

**DOI:** 10.1155/2022/4795307

**Published:** 2022-07-06

**Authors:** Yan Cheng, Tengwei Liao, Nailong Jia

**Affiliations:** ^1^Department of Nephrology, The Third People's Hospital of Zhengzhou, Zhengzhou 453000, Henan, China; ^2^The First Clinical Medical College, Guangzhou University of Chinese Medicine, Guangzhou 510405, China; ^3^Department of Radiology, The Second Affiliated Hospital of Hainan Medical College, Haikou 570311, Hainan, China

## Abstract

This study was aimed to explore the relationship between depression and brain function in patients with end-stage renal disease (ESRD) complicated with depression based on brain magnetic resonance imaging (MRI) image classification algorithms. 30 people in the healthy control group and 70 people in the observation group were selected as the research objects. First, the preprocessing algorithms were applied on MRI images. With the depression classification algorithm based on deep learning, the features were extracted from the capsule network to construct a classification network, and the network structure was compared to obtain the difference in the distribution of brain lesions. Different classifiers and degree centrality, functional connection, low-frequency amplitude ratio, and low-frequency amplitude were selected to analyze the effectiveness of features. In the deep learning method, the neural network model was constructed, and feature extraction and classification network were carried out. The classification layer was based on the capsule network. The results showed that the correct rate of the deep learning feature extraction network structure combined with the capsule network classification was 82.47%, the recall rate was 83.69%, and the accuracy was 88.79%, showing that the capsule network can improve the heterogeneity of depression. The combination of fractional amplitude of low-frequency fluctuation (fALFF), DC, and amplitude of low-frequency fluctuation (ALFF) can achieve the accuracy of 100%. In summary, MRI images showed that patients with depression may have neurological abnormalities in the white matter area. In this study, the classification algorithm based on brain MRI images can effectively improve the classification performance.

## 1. Introduction

With the continuous development of the economy and the continuous advancement of social development, China has also accelerated its entry into an aging society, and the number of people with poor sleep quality, anxiety, and depression is also increasing [1]. With the advancement of dialysis technology and the implementation of medical insurance, more and more end-stage renal disease (SRD) patients are receiving hemodialysis treatment, and their survival time is relatively longer [2]. According to relevant statistics, about 1.8%–23% of women and 1.1%–15% of men in the general population have depression, and the proportion of depression in ESRD is 20%–30% [3]. Depression seriously affects the physical and mental health of patients, and severely ill people will have thoughts of committing suicide. Physical complications and abnormal signs of ESRD patients, such as blood system, bone disease, and cardiovascular disease, have attracted special attention. An ESRD patient will have abnormal mental or neurological syndromes such as anxiety, depression, neurasthenia, and fear [4]. It is often easier to be ignored. Depression can cause the patient's mood or mood to be low, lack of interest, and suicidal tendencies, somatic symptoms such as sleep disturbance, appetite disturbance, sexual dysfunction, and non-specific somatic diseases such as pain and general discomfort. Depression generally affects the life of patients through the patient's concern for their own health, deterioration of nutritional status, abnormal immune function, medication and dialysis compliance, and low response to stress state [5, 6].

The etiology of depression in ESRD patients is caused by physical factors, social and psychological factors and other factors. Uremia toxins and complications can induce or aggravate the depression symptoms of ESRD patients. Marital relationship, economic pressure, and social support can also aggravate the depressive symptoms of ESRD patients [7, 8]. Diffusion tensor imaging (DTI) is a new type of diagnostic method developed on the basis of diffusion-weighted imaging, which can accurately describe the expansion direction and amplitude of water molecules. DTI can also show the path of white matter fiber tracts in vivo. The data obtained by DTI can be used to reconstruct the three-dimensional microscopic directional map of the white matter fiber tracts of the brain. This method of imaging is called diffusion tensor tractography (DTT) [9]. DTT imaging is a new technology developed on the basis of diffusion-weighted imaging. Six directions are added to the diffusion sensitivity gradient, and the diffusion state of water molecules is described based on the three-dimensional space. With the rapid development of imaging technology, DTT plays an important role in the diagnosis and treatment of brain diseases [10]. This imaging method is based on the similarity of the shape and direction of the diffuse ellipsoid between adjacent voxels. It is very useful for the analysis of the integrity and directionality of the central nerve fiber network [11].

At this stage, segmentation algorithms in the field of computer vision have many applications in the segmentation of image features, but they have not formed a sign of evaluating the quality of the algorithm. Machine learning based on computer intelligence is easier to recognize and analyze data [12, 13]. Intelligent segmentation of MRI image boundaries and features uses functional parameters to extract feature contours, and the image data obtained through image segmentation is more scientific, providing effective reference value for disease prediction [14, 15]. Compared with other medical images, MRI can provide doctors with more brain information based on the collected data and design different scan parameters to make a good diagnosis of the disease. The preprocessing of MRI images can largely eliminate morphological differences between individuals, as well as machine noise mixed in during the acquisition process and physiological noise caused by non-cerebral neural activities. The extraction of MRI image features can effectively describe the gray matter of the brain or brain neural activity from different angles, which is also a way to analyze and diagnose the brain structure of patients with depression [16, 17]. Brain MRI images of depression patients can clearly distinguish the difference between the two through MRI images, and pathological research on MRI with the help of machine learning methods can become an important content in computer-aided diagnosis [18].

In this study, the MRI images of patients with ESRD complicated with depression were firstly processed with algorithms, and then features were extracted. The classifier design and fusion algorithm from multiple perspectives were adopted to analyze the impacts of different characteristics on the brain function of patients, aiming to provide a reference for the treatment of patients with depression in the clinic.

## 2. Methods

### 2.1. Research Objects

In this study, ESRD patients who were hospitalized from June 2018 to December 2020 were selected and defined as the observation group. They all meet the diagnostic criteria of major depression in the American Diagnostic and Statistical Manual of Mental Disorders (4^th^ Edition). The specific items included (i) 18–68 years old, Han nationality, and right-handed; (ii) the total score was ≥18 evaluated using the 17^th^ edition of the Hamilton Depression Scale; (iii) before enrollment, the patients had not used antidepressant or antipsychotic drugs, and none of the patients had alcohol dependence, hypertension, diabetes, schizophrenia, and epilepsy, etc., There were 70 cases that met the inclusion criteria, including 26 males and 44 females; and their age ranged from 32 to 63 years old, with an average of 51.56 ± 2.74 years old. The patient's course of illness was 2–12 months, and the dialysis time was 7–10 months. There were 14 cases of polycystic kidney disease, 13 cases of chronic interstitial nephritis, 16 cases of obstructive nephropathy, 15 cases of hypertensive nephropathy, and 32 cases of chronic glomerulonephritis. The general condition of the patient was recorded in detail.

The inclusion criteria were defined as follows: patients who met the ESRD diagnostic criteria and aged ≥18 years old; patients with complaints of mental abnormalities, unconscious disorders, and sleep disorders; patients with good understanding and communication skills; and patients who were volunteer to join this study.

The exclusion criteria were given as follows: patients who had a history of severe mental disorders and those who did not cooperate; patients whose image analysis results were affected by factors such as serious image motion artifacts; patients who were unable to take care of themselves, were slurred in speech, were seriously ill, etc.,; patients who cannot cooperate with the investigation or who did not agree to participate in the investigation; and patients whose kidney DTI images showed renal abnormalities, changes around the kidney, and diseases of the collective system.

Healthy control group: employees and healthy volunteers were recruited. The inclusion criteria were given as follows: (i) no drugs had been used before enrollment; (ii) individuals and families had no current or ever suffered mental disorders;(iii) no obvious traumatic brain injury;(iv) no MRI contraindications; (v) no obvious medical disease; and (vi) the patient voluntarily participated in the study to understand the content of the study. 30 healthy people were studied, including 12 males and 18 females, aged 22–57 years old, with an average of 53.09 ± 2.38 years old.

This study had been approved by the ethics committee of the hospital. All patients and their families had signed the informed consent forms.

### 2.2. Assessment Using Scale

The Chinese version of the Hamilton Rating Scale for Anxiety (HAMA) (14^th^ version) was selected to evaluate the anxiety of the two groups of subjects. Both Chinese version scales were widely used in clinical and scientific research, and their validity and reliability were very good.

### 2.3. MRI Scan Image Parameters

In this study, all patients and healthy controls received 3.0T superconducting MRI whole body scanner, using 8-channel head coils for MRI scanning. Before the scan, we described the examination process in detail to the patients, made the patients supine, and advised them to use earplugs to reduce the impact of equipment noise and maintain steady breathing. The scanning sequence included T1 fast-recovery fast spin-echo (FSE) (FRFSE) sequence and DTI sequence. The same level and positioning were adopted, the positioning line was parallel to the anterior commissure-posterior commissure (AC-PC) plane, and the whole brain was scanned from the base of the skull to the top of the skull. The scan time was about 20 minutes. The scanning parameters were described as follows. For cranial horizontal axis FSE sequence T1WI: time of echo (TE) was 15–25 ms, and time of repetition (TR) was 500 ms. For FSE T2WI: the TE and TR were 90–120 ms and 2500 ms, respectively; layer thickness was 8 mm, and layer spacing was 2 mm. DWI scanning used a single excitation plane echo sequence, using 2 dispersion *b* values (0–1000 s/mm^2^): the field of view (FOV) was 24 cm × 24 cm, matrix was 256 × 128, layer thickness was 8 mm, layer spacing was 2 mm, three perpendiculars to each other dispersion sensitive gradient direction, and the TR/TE was 6000/110 ms. The DTI sequence scan also used a single excitation plane echo sequence, the horizontal axis scan, the dispersion sensitive gradient direction was 13, t and the TR/TE was 10000/110 ms. The FOV was 24 cm × 24 cm, matrix was 256 × 128, layer thickness was 5 mm, layer spacing was 0 mm, NEX = 1, and the scanning time was 160 seconds. The obtained MRI images were sent to the workstation, and the images were processed using Function II software.

### 2.4. Image Analysis

The data obtained from MRI scans were used in the software and 3.0T MRI scanner. Two experienced doctors with 5 years of working experience in the diagnostic imaging department were invited to select random double-blind principles to analyze the DTI images of patients. The diagnosing physicians read the film without knowing the general clinical data of the patients. First of all, the quality of the image was evaluated, and artifacts and noises in the image were removed to avoid affecting the doctor's diagnosis of DTI. The morphology and DTI images that met the diagnostic requirements were included in the final DTI image analysis, the ADC and FA of the kidneys were finally measured, and the abnormalities of the kidneys were observed.

### 2.5. Image Standardization

**Figure 1:**
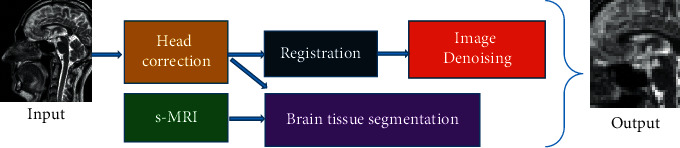
Schematic diagram of the standardization process of resting MRI images.

Image standardization was done to eliminate the differences in the morphology of individual brains and interference and analyze the fixed points of MRI images. The standardization process of MRI images is shown in Figure 1 below.

### 2.6. Feature Extraction of Image

**Figure 2:**
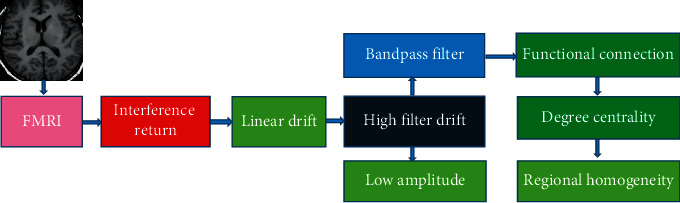
Feature extraction in preprocessing.

In the feature extraction of MRI images, the intensity of each voxel in the brain changed with time series. The changing information can be used to describe the brain nerve activity from different angles. The commonly used variable characteristics mainly included degree centrality, regional homogeneity, low-frequency amplitude, functional connection, and low-frequency amplitude ratio. These features can prove whether the brain nerve activity was normal, and these features were also different in the extraction stage of different preprocessing processes. The extraction stage of different features in the MRI image preprocessing process is shown in Figure 2.

Functional connectivity refers to changes in the brain with mental illness, which can reflect the increase or decrease of the connection between the brain function network areas. Different areas of the brain have different perceptions of functions such as hearing, cognitive control, emotional understanding, and vision. Researchers generally look at the brain as a complex network. Each area is a node of the brain network, and the connection node is the functional connection of the brain. Many researchers have produced maps of different brain regions, which are divided into 246 or 116 brain regions. After MRI images are standardized, automatic anatomical markings are made in the Montreal Neurological Institute (MNI) space, which are used more in research. Based on this, the brain regions were divided with automatic anatomical markers in this study. First, the average value of the signal hydroxyl groups was calculated at different moments in the brain regions, as shown in Figure 1.

(1) $$\begin{array}{c} Xi=\sum Xg. \end{array}$$

*Xi* represents the *i*th brain area and *Xg* represents the signal intensity of voxel *g* in the brain area, then, the Pearson correlation coefficient *f*^*ij*^ between the two brain areas is expressed as follows:

(2) $$\begin{array}{c} f^{ij}=\frac{\mathrm{cov}\left(Xi,Xj\right)}{\varphi \left(Xi\right)\varphi \left(Xj\right)}=\frac{\sum \left[\left(Xi\left[t\right]-\bar{X}i\right)\left(Xj\left[t\right]-\bar{X}j\right)\right]}{\sqrt{\sum \left[{\left.Xi\left[t\right]-\bar{X}i\right)}^{2}{\left(Xj\left[t\right]-\bar{X}j\right)}^{2}\right.}}. \end{array}$$

In equation (2), *Xi[t]* and *Xj[t]* represents the regional average signal intensity of brain area *i* and brain area *j* at time *t*, respectively; $\bar{X}i$ and $\bar{X}j$ refers to the time average signal intensity of brain area *i* and brain area *j* in the entire time series, respectively. The function connection matrix is as follows:

(3) $$\begin{array}{c} R_{c}=\left[\begin{array}{c} f_{11}f_{12}f_{13}\ldots f_{1N}\\ f_{21}f_{22}f_{23}\ldots f_{2N}\\ \ldots \\ f_{N1}f_{N2}f_{N3}\ldots f_{NN} \end{array}\right]. \end{array}$$

In the equation above, *N* represents the size of the matrix, and N also represents the number of brain regions in the map. If *N* = 116, the brain is divided into 116 brain regions and below equation is obtained:

(4) $$\begin{array}{c} f_{ij}=f_{ji}. \end{array}$$

Then, it is necessary to extract the functional connection of the triangular area in the matrix. The extraction of functional connection features still depends on the structure of the brain network Atlas.

### 2.7. Degree Centrality and Regional Homogeneity

Degree centrality represents the association between a given voxel and the whole brain voxel. If certain nodes in the brain are abnormal, some areas cannot be matched with healthy people's nodes consistently, which will lead to mental illness. In the contrast center feature extraction, the connectivity of the brain is measured by the number of strong correlation connections of brain voxels, and the correlation between different voxels is expressed as an equation.

(5) $$\begin{array}{c} f_{ij}=\frac{\sum \left[\left(X_{i}\left[t\right]-{\bar{X}}_{i}\right)\left(X_{j}\left[t\right]-{\bar{X}}_{j}\right)\right]}{\sqrt{\sum \left[{\left.X_{i}\left[t\right]-{\bar{X}}_{i}\right)}^{2}{\left(X_{j}\left[t\right]-{\bar{X}}_{j}\right)}^{2}\right.}}. \end{array}$$

*X*_*i*_*[t]* and *X*_*j*_*[t]* represents the regional average signal intensity of brain area *i* and brain area *j* at time *t*, respectively; and the average time voxel signal intensity of the *i*th and *j*th voxel positions are denoted as $\bar{X}i$ and $\bar{X}j$, respectively.

If there were *n* voxels in the MRI image, an undirected unweighted adjacency matrix of *n∗n* size can be constructed according to the coefficients, and the centrality measure of voxel *i* is shown in the following equation:

(6) $$\begin{array}{c} d_{ij}=\left\{\begin{array}{c} 1f_{ji}>0.25,i\neq j,\\ 0\text{other,} \end{array}\right. \end{array}$$

(7) $$\begin{array}{c} Db_{ij}=\sum d_{ij},j=1,2,\ldots N,i\neq j. \end{array}$$

The correlation coefficient between voxels *ij* in equation (6) represents the number of other voxels connected with each voxel, and the feature is extracted.

Regional homogeneity is the abnormality of neural activity on the basis of brain lesions, which reflects the degree of synchronization of the voxel field signals of the FMRI data in the time series. Feature extraction itself has a smoothing effect and did not require Gaussian filtering. Feature acquisition was achieved by calculating voxels and neighboring voxels. The equation is as follows:

(8) $$\begin{array}{c} Q_{i}=\frac{\sum_{i}X_{i}{\left[t\right]}^{2}-N\sum_{i}X_{i}{\left[t\right]}^{2}}{\left(1/12\right)v\left(N^{3}-N\right)}. \end{array}$$

*X*_*i*_*[t]* represented the voxel intensity at the *i*th voxel position at time *t*, *V* represents the number of selected domain intensity voxels, and *N* reached the total number of voxels.

Amplitude of low-frequency fluctuation (ALFF) can be used to detect the difference in neuronal activity between depressed patients and healthy people. When the low-frequency amplitude was calculated, the image was band-pass filtered. The energy range of the filtered signal was (0.001–0.08), and the frequency spectrum calculation equation is as follows:

(9) $$\begin{array}{c} X_{i}\left[h\right]=\sum_{i=1}^{n}X_{i}\left[k\right]e^{j\left(2\pi /N\right)hk}. \end{array}$$

*X*_*i*_ represents the frequency spectrum calculated by the Fourier transform of the time series of voxel *i*.

The low-frequency amplitude of the voxel is calculated as follows:

(10) $$\begin{array}{c} ALEE_{i}=\sum_{K}\sqrt{X_{i}{\left[k\right]}^{2}},K\in \left[0.01\frac{2\pi }{f_{s}^{2}},0.08\frac{2\pi }{f_{s}^{2}}\right]. \end{array}$$

In equation (10), fs represents the sampling frequency.

The feature extraction of fALFF was based on the problem that the low-frequency amplitude of the physiological noise signal in the periphery of the large blood vessel, and the brain cistern was higher than the low-frequency amplitude of the brain nerve signal. The ratio of the total amplitude of the low frequency to the total amplitude of the entire frequency range is as follows.

(11) $$\begin{array}{c} FALEE_{i}=\frac{\sum_{K}\sqrt{X_{i}{\left[k\right]}^{2}}}{\left(1/N\right)\sum_{h=0}^{N-1}\sqrt{X_{i}{\left[k\right]}^{2}}},K\in \left[0.01\frac{2\pi }{f_{s}^{2}},0.08\frac{2\pi }{f_{s}^{2}}\right]. \end{array}$$

The low-frequency amplitude can suppress the physiological noise brought by the large blood vessels. When the low-frequency amplitude was close to the high-frequency amplitude, the equation (11) can inhibit it.

### 2.8. Classification Algorithm Based on Deep Learning

**Figure 3:**
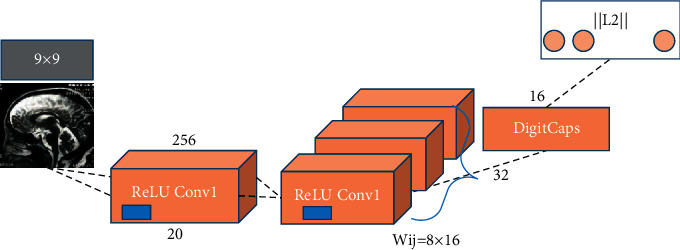
Classification algorithm of CapsNet.

Many clinical MRI images in the study of patients with depression have large feature dimensions, and the disease response of mental disorders exists in all corners of the brain. In some areas, there are a large number of initial features that do not interfere with each other. To extract features in a very large space, a good classifier is needed to select the optimal feature subset. In the designed classifier, the classification model is used to classify new samples. In the deep learning algorithm, the Capsule Network (CapsNet) is an emerging neural network architecture. Compared with the convolutional neural network (CNN), the CapsNet has stronger generalization ability and does not require a lot of training. Some precise details can be better preserved, such as the position, inclination, thickness, and size of the object's rotation. It can also enhance the feature extraction ability of network details without an additional process of losing and then recovering. The capsule network can be simple a unified architecture completes different visual tasks. As shown in Figure 3, after a standard convolutional layer ReLU Conv1 with a step size of 1 was inputted, it would generate 256 channels for feature images. Then, a modified capsule convolutional layer was inputted to generate an 8-dimensional vector instead of a table volume. The length of each vector ||L2|| represents the possibility that the input image belongs to 10 categories.

The optimization of network parameters is different from general neural networks. The CapsNet uses dynamic routing to back-propagate and iteratively update the parameters of each layer. The calculation equation of the loss function is as follows:

(12) $$\begin{array}{c} L_{p}=T_{p}\max\;{\left(0,m^{+}-\left\|V_{p}\right\|\right)}^{2}+\lambda \;\left(1-T_{p}\right)\max\;{\left(0,\left\|V_{p}\right\|-m^{-}\right)}^{2}. \end{array}$$

In the above equation (12), *P* represents the classification category index, *m*^*+*^ and *m*^*−*^ takes values 0.9 and 0.1, respectively. When the sample type is *P*, when *T*_*P*_ is set to 1, the loss function is expressed as follows:

(13) $$\begin{array}{c} L_{p}=\max\;{\left(0,m^{+}-\left\|V_{p}\right\|\right)}^{2}. \end{array}$$

The value of *T*_*P*_ is close to 0.9, and the loss function is close to 0, which is also the model result that the network hopes to train.

If the sample category is not P, the loss function is given as follows:

(14) $$\begin{array}{c} L_{p}=\lambda \max\;{\left(0,\left\|V_{p}\right\|-m^{-}\right)}^{2}. \end{array}$$

The appearance of the *λ* value reduced the loss value that did not appear in the *P* category, and thus, avoiding the initial loss from being too large, resulting in the shrinking of the length of all output vectors. The overall loss can be expressed as below equation (15):

(15) $$\begin{array}{c} L^{\left(i\right)}={\sum_{p}L_{p}}^{\left(i\right)}. \end{array}$$

### 2.9. Statistical Methods

SPSS21.0 statistical software was adopted to perform statistical analysis on DTI parameters. Measurement data conforming to the normal distribution were represented by mean ± standard deviation (‾*x* ± *s*), and non-conforming count data were represented in the form of frequency or percentage (%). *α* = 0.05 was undertaken as the test level for comparison between groups. When *P* < 0.05, the difference was considered to be statistically significant.

## 3. Results

### 3.1. General Data of Patients

The comparison of the demographic data of the two groups of study subjects showed that there was no statistical difference between the healthy control group and the depression group in terms of gender, education level, age, and marital status (*P* < 0.05) (Table 1).

The general information of the observation group is shown in Table 2. The main occupations were workers, farmers, civil servants or institutions, self-employed, and teachers. The main sources of expenses were municipal medical insurance, provincial medical insurance at their own expense, and rural cooperative medical care.

### 3.2. Network Classification Results

**Figure 4:**
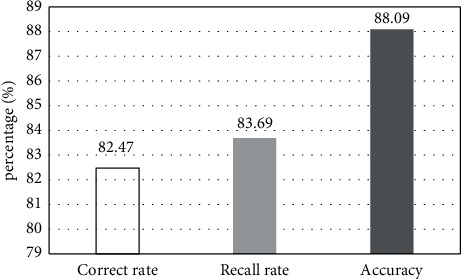
Network classification results.

The neural network model was constructed in the deep learning method, and the feature extraction and classification network were performed. The classification layer was based on the capsule network. The experimental results of the CNN feature extraction network structure and the CapsNet classification are shown in Figure 4. The correct rate of the constructed network was 82.47%, the recall rate was 83.69%, and the accuracy was 88.79%. It showed that the CapsNet can improve the heterogeneity of depression, which was suitable for the classification of MRI images.

### 3.3. Multi-Feature Ensemble Classification Results

**Figure 5:**
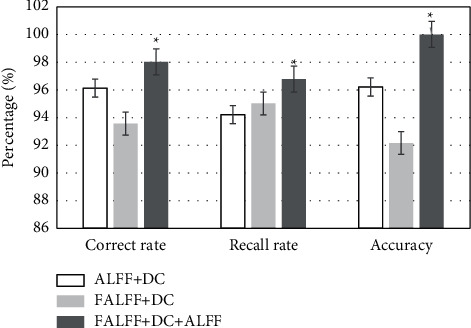
Integrated classification results. ^∗^compared with the other two groups, ^∗^*P* < 0.05.

The idea of ensemble learning is to integrate multiple feature classification models to classify depression. In order to maximize the classification performance, fALFF, DC, and ALFF were aggregated, and the prediction results of the combined model were weighted and averaged to obtain the final classification result. It can be observed from the results shown in Figure 5 that the combination of the three can achieve the accuracy of 100%. Compared with the other two forms, the combination of the three had significant difference (*P* < 0.05).

### 3.4. MRI Images

**Figure 6:**
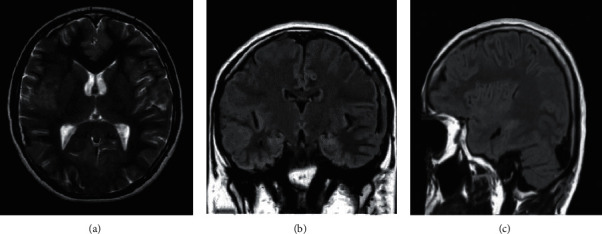
MRI images of healthy people.

The healthy patients were selected to observe MRI images.  Figure 6 shows the images on MRI showing no obvious structural abnormalities.  Figure 6(a) is a cross-sectional MRI image of the main structures of the brain, which can show structures such as the interhemispheric fissure of the frontal lobe and the superior cerebral vein.  Figure 6(b) shows a normal axial image of the brain lobes, with the frontal lobe occupying 2/3 of the upper layer of the cerebral hemisphere, and the occipital lobe near the posterior horn of the lateral ventricle.  Figure 6(c) shows a craniocerebral anomaly, clearly showing the middle cerebral artery in the middle frontal gyrus of the craniocerebral gyrus and in the center of the semiovale.

**Figure 7:**
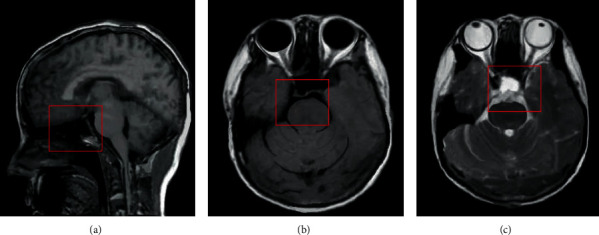
MRI images of a patient.

Figure 7 shows an image of a male patient in the observation group.  Figure 7(a) is a side view of the brain, and an MRI image shows a disorder of the central nervous system. Figures 7(b) and 7(c) show the “tiger stripe sign”, T1WI shows a streak-like structure with low signal in the high-signal area, and the MRI signal shows abnormal lobular transverse shape. The lesions are mainly concentrated in the hypothalamus, and there are abnormalities in the hypothalamus pituitary gland in the sellar region. The arrow in the figure marks the location of the lesion. Butterfly pituitary stalk moved backward with uniform signal strength. The red arrows in Figure 7 indicat the location of the lesions.

### 3.5. Registration Image of  MRI  Image

**Figure 8:**
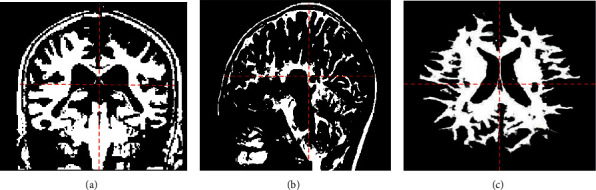
MRI image registration example.

The images in Figure 8 are from a 52-year-old male patient. After the image was processed by the algorithm, the definition was higher. The blue line in the figure shows the process of image registration by the instrument. After the objective function was optimized, the image registration was performed according to the selection of the parameters, and the example effect is shown in Figure 8. The image was registered from the structural master image.  Figure 8(a) shows that the registration line is parallel to the line connecting the frontal lobes on both sides.  Figure 8(b) shows the parallel anterior fossa floor.  Figure 8(c) shows where the configuration line is located in the frontal lobe.

### 3.6. Comparison of White Matter FA

The anisotropy score (FA) was used to quantitatively measure the size of the third anisotropy of water molecules in the white matter fiber bundles. It was positively correlated with the integrity of the myelin sheath, the compactness and parallelism of the fibers, and can reflect the completeness of structure of the white matter. Compared with the healthy group, the main manifestations of the observation group were the right posterior cingulate gyrus, the right lingual gyrus, the right prefrontal lobe, the medial marginal lobe of the right upper gyrus, and the right talus gyrus. The specific results are listed in Table 3.

## 4. Discussion

ESRD is a disease that cannot be cured. Patients need to rely on dialysis to maintain their lives. During the entire treatment process, the patient's psychology will produce various emotional distress.

Relevant studies have shown that 50%–80% of ESRD patients have different types and degrees of sleep disorders. Sleep disorders and anxiety in patients with depression complement each other and promote each other. The two have good compliance. People with severe depression sometimes have suicidal tendencies, which increase the economic burden of society and families [19]. Health status affects the quality of life of patients, and what they want to do produces resistance in thought and life. Family support can improve patients' depression and anxiety caused by dialysis, and effectively improve patients' treatment compliance. Yoong et al. [20] found that 49.9% of ESRD patients experienced increased depression, and 45.4% of de-ESRD patients experienced increased anxiety during treatment. The high proportion of anxiety and depression emphasized the importance of detection and care for ESRD patients during dialysis care. Reckert et al. [21] reported that depressive symptoms are relatively common in ESRD patients and have a great correlation with morbidity and mortality. Depression and anxiety symptoms are related to the gender, employment status, and physical activity of ESRD patients. Anxiety symptoms are also closely related to body mass index, and physical activity can be used as a protective factor for patients with ESRD. In the study, workers accounted for the highest occupational proportion of ESRD patients (40%), and the least proportion was teachers, the proportion was 10%. The number of patients in each occupation is different, and the emotions generated are different, which may be related to the nature of the job itself.

As a non-invasive imaging method, DTI can reflect the directional diffusion of water molecules in the tissue by measuring partial anisotropy, and it can also evaluate the translation of water molecules in various directions by measuring ADC [22, 23]. MRI images of the brain of patients with depression showed that the patients' hippocampus, insula, and amygdala were abnormal, and the frontal cortex, cingulate gyrus, hippocampus, and striatum of unidirectional depression were reduced in volume [24]. In recent years, studies have confirmed that the introduction of intelligent algorithms, such as deep learning, into medical images has a great effect on image quality improvement, lesion recognition, and extraction and segmentation [25]. It was found in this study that the FA value decreased, and the spatial attention and memory of depression patients decreased.

## 5. Conclusion

In this study, based on the classification algorithm of deep learning, a network structure was constructed to extract and classify patient MRI images. In the deep learning method, a neural network model was constructed to perform feature extraction and classification network. The classification layer was based on capsule network with high accuracy. The algorithm of this study could effectively classify the features of the concave region. Research on classification algorithms based on brain MRI images requires more research, and the number is bound to increase. In the future, more good algorithms should be effectively verified to provide reference for computer-aided diagnosis in clinical practice.

## Figures and Tables

**Table 1:** Comparison on general data of patients in two groups.

Item	Depression group	Healthy control group	*P* value
Number of cases	70	30	—
Age (years old)	51.56 ± 2.74	53.09 ± 2.38	—
Gender	—	—	0.574
Number of males	26	12	—
Number of females	44	18	—
Marital status	—	—	0.879
Unmarried	14	7	—
Married	56	23	—
Education level (years)	12.24 ± 3.65	15.24 ± 2.61	0.243
HAMD (scores)	26.37 ± 4.36	3.37 ± 1.13	—
HAMA (scores)	21.43 ± 5.21	2.41 ± 1.42	—

**Table 2:** General data of patients.

Category	Number	Profession	Number	Average age	Source of expenses	Number
Polycystic kidney	14	Worker	26	50.06 ± 3.13	Own expense	8
Chronic interstitial nephritis	13	Farmer	12	42.60 ± 4.04	District medical insurance	8
Obstructive nephropathy	11	Civil servants	8	31.56 ± 5.78	City medical insurance	11
Hypertensive nephropathy	10	Self-employed	15	46.56 ± 2.28	Social medical insurance	14
Chronic glomerulonephritis	22	Teacher	9	31.06 ± 3.62	Rural cooperative medical	29
Total	70	—	70	51.56 ± 2.74	—	—

**Table 3:** Comparison of white matter FA between the two groups.

Brain area with decreased FA value	Voxel (mm^2^)	Z	T	*P*
Right posterior cingulate back	14	3.425	3.912	<0.01
Right lingual gyrus	23	4.501	3.822	<0.01
Right frontal lobe	31	3.921	3.401	<0.01
Medial marginal lobe of upper right side	48	3.465	3.612	<0.01
Right talar gyrus	121	3.987	3.608	<0.01

## Data Availability

The data used to support the findings of this study are available from the corresponding author upon request.
